# Undesirable Postoperative Anesthesia Outcomes at Two National Referral Hospitals: A Cross-Sectional Study in Eritrea

**DOI:** 10.1155/2020/9792170

**Published:** 2020-09-15

**Authors:** Yonatan Mehari Andemeskel, Traudl Elsholz, Ghidey Gebreyohannes, Eyasu H. Tesfamariam

**Affiliations:** ^1^Department of Anesthesia and Critical Care, School of Nursing, Asmara College of Health Sciences, Asmara, Eritrea; ^2^Asmara College of Health Sciences, Asmara, Eritrea; ^3^Department of Epidemiology and Biostatistics, School of Public Health, Asmara College of Health Sciences, Asmara, Eritrea

## Abstract

**Background:**

Postoperative undesirable anesthesia outcomes are common among patients undergoing surgery. They may affect body systems and lead into more serious postoperative problems. This research is conducted in the Eritrean National Referral Hospitals with the aim of assessing the prevalence of undesirable anesthesia outcomes during the postoperative period.

**Method:**

A cross-sectional study design was applied on 470 patients who underwent different types of surgeries within a three-month period. Patients were interviewed 24 hours after operation (POD 1) using the Leiden Perioperative care Patient Satisfaction questionnaire (LPPSq). This study reports one component of a large study conducted. The dimension “Discomfort and needs” of the LPPSq was considered, and the measurements of that dimension are presented in this report. Items of the dimension were standardized and measured using a five-point Likert scale from “Not at all” to “Extremely.” Multivariable logistic regression was used to look for the association of the outcomes with the types of surgery and types of anesthesia using SPSS (Version 22).

**Results:**

The prevalence were computed in two manners, prevalence of those with ‘at least a little bit' outcomes, which was computed to see the total occurrence of these outcomes, and prevalence of those having ‘more than moderate' outcomes to see the severe experience of these outcomes. Prevalence of the predominant undesirable outcome, postoperative pain, for ‘at least a little bit' and ‘more than moderate' were 82.6% and 43.6%, respectively. The rest of the postoperative undesirable outcomes were less frequently reported.

**Conclusion:**

Postoperative pain was found to be the most prevalent undesirable outcome. Enhancement of proper assessment and management of postoperative pain through the development and implementation of specific pain management modalities is needed.

## 1. Background

Undesirable postoperative anesthesia symptoms are common and may affect all patient's body systems [1]. The most commonly mentioned complications are pain, nausea, vomiting, sore throat, shivering, thirst, and hunger [1–3]. These complications has also been observed to trouble patients from the Eritrean National Referral Hospitals during their postoperative stay. Underestimation of these undesirable events and lack of adequate management protocols and skills persist to enhance their occurrence.

Postoperative pain is a common experience for postoperative patients and remains a serious problem facing anesthesia providers in their daily practice [4]. Poor management of acute postoperative pain is among the causes for some medical complications, and it prolongs the time of recovery and hospitalization increasing postoperative morbidity [5, 6]. Although the authors could not find any study to demonstrate the quality of postoperative pain management at the level of patients, pain is undervalued, and insufficient knowledge of pain management persists. As an acute care management, diclofenac as an intramuscular injection is the commonly prescribed drug followed by the orally taken NSAIDs. Opioids are hardly utilized, associated with the lack of knowledge, general fear of utilizing opioids, and most importantly shortage of availability. Moreover, postoperative pain medications are still prescribed on as needed basis requiring patients to ask for pain medication, and treatment is usually provided when patients experience severe pain. Meanwhile, analgesia should be the fundamental right of every patient, and allowing patients to experience postoperative pain is unacceptable and unethical especially when tools and educated health care providers are available [7]. Proper postoperative pain management significantly enhances recovery and reduces patient morbidity increasing the hospital stay and cost [6, 8]. Thorough evaluation and objective measuring of pain with the pain measuring scales such as the visual analog scale is important as it helps in determining the effectiveness of treatment [9–11]. Postoperative nausea, vomiting, and hypothermia are also among the frequently occurring undesirable events [12, 13]. Generally, failing to prevent these undesirable events have been linked to subsequent, more serious postoperative problems [2]. They have also been explained as modifiable sources of patient dissatisfaction [3].

The effectiveness of managing these undesirable events is determined by the correct and timely detection of the symptoms and applying appropriate pharmacotherapy [1]. Recognizing and treating these complications is vital in the provision of good quality of care [1, 2]. Meanwhile, it has been observed in the study settings that these undesirable postoperative events to be common troubling postoperative patients, increasing their morbidity and prolonging their recovery and hospital stay. No local guideline or management protocol exists for the management of these undesirable events. A patient who undergoes anesthesia is at risk of complications, and the anesthetist is expected to be responsible in securing patient safety through adequate management of these risks and outcomes [14, 15]. Meanwhile, in Eritrea, the work of anesthetists is usually confined only to the operating room. No anesthetist is involved in the management of patients during their postoperative stay unless the patient undergoes in to an unexpected condition that requires emergency management. Hence, meaningful anesthetic evaluations and management of postoperative patient outcomes are hardly a practice in these settings. This study, the first of its kind in the country, is therefore aimed at determining the prevalence of anesthesia-related postoperative undesirable outcomes among patients undergoing surgery in the selected hospital settings. The results could reliably show areas of intervention to improve the quality management of the postoperative undesirable events be discussed, and effective management protocols could be formulated consistent with the international guideline that would optimize the management of these undesirable events so as to improve the quality of care. The results will also extend the body of literature and serve as a baseline for further studies.

## 2. Methods

### 2.1. Study Design and Setting

This descriptive, cross-sectional study was conducted between January and March of 2018 in Eritrea, a country in the horn of Africa. Eritrea has two National Referral Hospitals, which are located at the Capital city, Asmara. They are called Halibet and Orotta National Referral Hospitals and both of them provide health services at a tertiary level. They are the only governmental medical surgical national referral hospitals in which all types of major and minor surgeries take place.

### 2.2. Participants

During the study period, a total of 526 patients underwent surgeries under general and regional anesthesia. Respondents' eligibility was based on their willingness to participate in the study. Patients under the age of 18 years, those who were discharged before 24 hours of postoperative period, patients with serious illness, and those who did not consent to participate were excluded from the study, and the final sample size was 470.

### 2.3. Data Collection Tool and Method

The key elements of sociodemographic and clinical characteristics of the patients were obtained using a sociodemographic and clinical form. The sociodemographic and clinical details obtained were age, gender, place of residence (urban or rural), occupation, hospital setting, health coverage, type of anesthesia, type of surgery, and admission type (emergency or elective). The undesirable anesthesia outcomes were measured using the dimension “discomfort and needs” of the Leiden Perioperative care Patient Satisfaction questionnaire (LPPSq). The LPPSq was initially modified by Calijouw et al., and permission was asked and obtained from the responsible author. The LPPSq is a validated suitable research scale [16], having six dimensions, in which ‘discomfort and needs' was separately handled and analyzed because of its unique psychometric characteristics [17, 18]. As far as the founders of the scale are concerned, the internal consistency within the discomfort and need dimension was so low that it was not incorporated to get a composite score of the LPPSq, resulting to analysis of the individual items separately. This of course was one of the clear indications that the items listed in the dimension provide wider objectives and another perspective of anesthesia service. Explanation of the five other dimensions is made in previous publication [19]. The ‘discomfort and needs' investigates the common adverse outcomes of anesthesia raised from patient's perspective including postoperative pain, sore throat, back pain, nausea, vomiting, cold, hunger, thirst, and headache.

The study was conducted by independent researches who were not involved in any of the perioperative anesthetic or any other postoperative management of patients in the study settings, and data were collected through face to face interview by four well trained anesthetists who do not work in the study settings. They were also assistant researches and were well aware of the study objectives.

### 2.4. Data Collection Procedure

The researchers visited each hospital and explained the purpose of the study and its clinical significance to the hospital directors after getting the ethical clearance approval by the Research and Human Resources Development, Ministry of Health. Permission to conduct the study was then obtained from each hospital director. Recruitment of the patients was undergone before the moment of their operation. After full explanation of the study objectives and assurance of confidentiality and anonymity, patients were given written informed consent. The interview was then conducted in their respected postoperative wards after assuring that they felt comfortable for the interview, and the time to complete the questionnaire was about 15–20 minutes.

### 2.5. Variables and Measures

The items in the ‘discomfort and need' were standardized and measured using a five-point Likert scale. Patients had to state to which degree they experienced each of the attribute stated in each item after operation. The replies to the items were “Not at all” (0), “A little bit” (1), “Moderately” (2), “Quite a bit” (3), and “Extremely” (4). The items were then computed into two sequences of prevalence, prevalence of those with ‘at least a little bit' undesirable anesthesia outcome occurrence, which was computed to see the total occurrence of these undesirable outcomes, and prevalence of those having ‘more than moderate' postoperative undesirable anesthesia outcome occurrences to see the severe occurrence of these outcomes.

### 2.6. Validity and Reliability

Content validity of the items of the dimension was checked along with the rest of the dimensions by an expert' opinion from the anesthesia department. Translation (to the local language) was done by a bilingual language expert and was then back translated to English by another bilingual person who was not aware of the study objectives. A pretest was also performed to ascertain the comprehension and understandability of the questions. Internal consistency of the dimension was also checked (Chronbach *α* = 0.66).

### 2.7. Data Analysis

Responses were coded and entered into SPSS (Version 22) statistical software for analysis. Data cleaning and preliminary explorations were performed to assure accuracy of entry before conducting the main analysis. Frequency (percentage) and mean (standard deviation) were used to summarize the demographic and clinical variables of the participants. The prevalence of those with ‘at least a little bit' outcome occurrence and prevalence of those with ‘more than moderate occurrence' were also computed. Moreover, odds ratios (95% CI) were computed to assess the association of undesirable anesthesia outcomes with the types of surgery and types of anesthesia using multivariable logistic regression. *P* values less than 0.05 were considered as significant throughout the analyses.

## 3. Results

### 3.1. Population Characteristics

**Figure 1:**
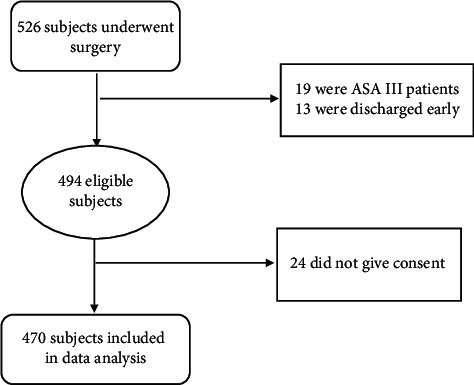
Eligible number of patients for the study.

A total of 526 patients underwent surgeries under general and regional anesthesia out of which 470 fulfilled the inclusion criteria and were included in data analysis.  Figure 1 shows a summary of the ultimate number of participants and their eligibility.

The demographic and clinical details of the participants are shown in Table 1. The age of the respondents ranged from 18 to 85 years, with a mean ± SD value of 45.9 ± 14.7. From the total 470 participants, 55.1% were males and 44.9% were females. The majority (63.2%) of the patients were from Orotta Hospital. The patients underwent a wide range of surgical procedures, including general, orthopedic, Gyn/obs, ENT, and burn surgery. 267 (56.8%) patients had general anesthesia and 203 (43.2%) had regional anesthesia.

### 3.2. Undesirable Postoperative Anesthesia Outcomes

Table 2 shows the prevalence with ‘at least a little bit' and ‘more than moderate' undesirable anesthesia outcomes. The predominant undesirable anesthesia outcome was postoperative pain, followed by cold, nausea, vomiting, thirst, back pain, headache, sore throat, and hunger. The prevalence of ‘at least a little bit' and ‘more than moderate' postoperative pain were 82.6% and 43.6%, respectively.

### 3.3. Association of Outcomes with Types of Surgery and Types of Anesthesia

As shown in Table 3, the association of the postoperative undesirable anesthesia outcomes with the types of surgery the patients underwent and the types of anesthesia those patients took were computed.

Significant difference in the occurrence of nausea and vomiting occurred in those patients who did general surgery. The odds of nausea (OR = 1.74, 95% CI: 1.04, 2.90) and vomiting (OR = 2.03, 95% CI: 1.19, 3.47) were higher among patients who took general anesthesia as compared to those who took regional anesthesia. On the other hand, significant experience of back pain was found in those patients who took regional anesthesia with the scores of 51% (OR = 0.49, 95% CI: 0.27, 0.88) in those who did general surgeries and 67% (OR = 0.33, 95% CI: 0.14, 0.80) in those who did Gyn/obs surgeries.

## 4. Discussion

This is the first survey in Eritrea that discusses about undesirable postoperative anesthesia-related events. It focuses on the most common undesirable postoperative anesthesia outcomes raised from patients' perspectives. One thing that should be considered is that the findings should be interpreted and understood within the context of the barriers of pain management being a developing country in which there is no national guideline that exist for the management of pain.

Generally, the management of these events is usually underestimated and is suboptimal, especially when it comes to the management of postoperative pain. The involvement of the anesthetist is very important in the management of these events. Moreover, having a local standard guideline is an important strategy in promoting standardization of procedures and patient controlled management. However, these two factors are not yet in practice in the study settings. The work of the anesthetist does not go beyond the operating room, and the management of these events is left either to the surgeon responsible or to the postoperative nurses. Moreover, unstandardized management protocol, shortage of experts in pain management, lack of adequate pain medications, and lack of knowledge to utilize the available ones adequately are some of the influencing factors.

The provision of adequate pain relief after surgery is an ethical responsibility of healthcare providers and a fundamental right of every patient [20–22]. Its importance has been emphasized during the early stages of recovery [23, 24]. Effective management of postoperative pain is an essential component in the provision of good quality of care, and it can have a significant effect on patient recovery by improving clinical outcomes, avoiding clinical complications, and finally saving health care resources [25, 26]. Not only during the postoperative period but adequate pain management plan requires preoperative preparation, up until after discharge to control pain effectively [27]. A preadministered analgesia reduces analgesic requirement [28]. Moreover, the accuracy in pain assessment has a major role in measuring the adequacy of pain management [4, 29]. Despite such an importance and its impact on the patient, postoperative pain is usually undertreated and suffering from pain continues to be a significant challenge [30–33]. It has also been explained that appropriate postoperative pain management is generally neglected in Eritrea [19]. Lack of skilled professionals and pain management guidelines also contribute to the condition, and pain management is more or less derived from the experience of the staff. In reflection to this, the experience of postoperative pain was found to be high in which the total experience of pain was 82.6%, of whom 43.6% experienced more than moderate pain. Not only in this study but also in the studies performed using the same questionnaire, postoperative pain was among the most frequently experienced complaints [17, 18, 34, 35]. Similar results were also reported in another study from the Netherlands by Kalkman and colleagues. This Dutch study was conducted in a university hospital on 1416 patients undergoing various surgical procedures with the aim of developing a validated prediction rule for the occurrence of early postoperative severe pain in surgical patients, and the incidence of severe pain was found to be 25.8%, measured one hour after surgery [36]. Another similar result was also reported in a study performed in Saudi Arabia. The study was conducted on 199 patients who did surgeries in a university hospital. The same questionnaire (LPPSq) was used, and they found postoperative pain to be the most frequently mentioned complaint [34]. Moreover, in a recent study performed in Mexico, the frequency of pain from moderate to severe level was determined to be 66.3% in which they concluded that their study represents ineffective pain management practices [30]. Worst pain experience score was also reported among 79% of the postoperative surgical patients in Ethiopia [37]. In the studies performed by Jlala et al and Calijouw et al., which were conducted with the primary aim of validating the LPPS questionnaire, thirst was another frequently mentioned complaint, unlike in this current study. The other postoperative side effects (back pain, headache, hunger, nausea, and vomiting) were less frequently reported with the lowest median score in all the abovementioned studies including this current study. Whereas according to a study performed in Rwanda, which was once again conducted using the same LPPS questionnaire on 145 patients, thirst and hunger were highly reported [35]. The status of postoperative pain was also compared between the two types of anesthesia; however, no significant difference was scored in its occurrence. Unlike in this study, in a study performed by Caljouw et al., 2008, a significant difference was observed in the occurrence of postoperative pain between general anesthesia (82.1%) and regional anesthesia (34.3%).

One of the first unpleasant symptoms in the postoperative period is postoperative nausea and vomiting [1, 38]. Despite anesthetic and pharmacological advances, there is no drug that is completely effective in preventing postoperative nausea and vomiting (PONV). They appear frequently, and they can often be complex and can be a significant problem in anesthesia practice. It is also often described as to be the second most common complaint after pain during the postoperative period [38, 39]. Understanding the mechanism and careful assessment of risk factors help in its management [2, 39, 40]. Normally, the occurrence of these unpleasant experiences is seen more commonly related to occur with general anesthesia than when using regional anesthesia [2, 12, 13, 41]. Similarly, in this current study, the occurrence of nausea and vomiting was higher among those patients who took general anesthesia. Since postoperative pain is commonly managed with NSAIDs and narcotics are hardly utilized for such purposes, it can be stated that the experience of nausea and vomiting is anesthesia related. As general anesthesia involves manipulation of the airway, it is obvious that the occurrence of sore throat to be higher in those patients who took general anesthesia. The very few sore throat that occurred among those who took regional anesthesia may not be purely associated with the anesthetic procedure. Moreover, the type of anesthesia given to all those who took regional anesthesia was spinal, and thus, the experience of back pain was high among these groups. Hypothermia affects more than 70% of patients undergoing surgery and anesthesia [13]. The main factors which lead to hypothermia include exposure to cold temperature during the intraoperative period, administration of unwarmed intravenous fluids, and evaporation from within the surgical incision [2, 42]. The deactivation of the thermoregulation center by general anesthesia itself can also lower the core temperature. Moreover, the usage of muscle relaxants during the operation greatly affects the patient's muscles ability to shiver and produce heat thus resulting in temperature drop [42]. This would be more pronounced in settings with limited resources like the operating rooms in the selected Eritrean study settings that otherwise would help to maintain the temperature of the rooms and of that of the patient. Meanwhile, the occurrence of hypothermia among the selected patients was not significant, and neither there was a significant difference in the occurrence of the rest of undesirable anesthesia outcomes such as hunger, thirst, and headache. Generally, postoperative undesirable events still persist despite the medical advances. There is a need to improve the treatment of these events by administering effective methods through an organized and systematized care, which is consistent with each setting. Such an approach would facilitate patient's recovery and shortens the hospital stay and will have a positive impact on patient's outcome and wellbeing [30].

Preoperative assessment is an important component of anesthesia [43]. Preparing a patient for anesthesia requires an understanding of the patient's preoperative status, the nature of the surgery, the anesthetic techniques required for surgery, and the risks that a particular patient may face during this time [44]. Problems with adequacy of preoperative assessment in the study settings, which was mentioned in a previous publication from this study [19], may also contribute to the increment in the prevalence of these undesirable outcomes. Patients should be provided with adequate information of the possible postoperative outcomes during the preoperative assessment, and depending on the results of this study, techniques of managing postoperative pain should be considered and discussed starting from the preoperative period. Postoperative care should be delivered by a multidisciplinary team that includes the anesthetist to gain a better outcome. It is very important to scale the level of pain so as to provide a management that corresponds with the level of severity. The type of surgery and type of anesthesia should also be considered. Narcotic agents alone can be given in conjunction with regional anesthesia, and their effect may also be extended by continuing the treatment with NSAIDs. This would reduce the undesirable effect from the narcotics and enhance postoperative pain management.

### 4.1. Limitations of the Study

Some of the exclusion criteria may have impacted the results of the study and the fact that the participants were made to be interviewed before their discharge, and the dependence of care might have retrained them from speaking their mind.

## 5. Conclusion

Postoperative pain was the most predominant undesirable anesthesia outcome. This gives a signal for the study settings to consider the involvement of anesthetists or dedicated pain nurses in the management of postoperative pain. This would enhance the proper management of postoperative pain through the development and implementation of specific pain management modalities. Local guidelines should be prepared in these settings to use for the treatment of acute postoperative pain. Moreover, measuring anesthetic outcomes in a reliable and valid manner would also be important for improving the standards of anesthetic care and delivering the quality of anesthesia in these settings. It has also been reported that adequate information is not provided to patients regarding their postoperative experience. Therefore, it is required for the study settings to make efforts on providing consistent preoperative information regarding choices for anesthesia, the risks and benefits of the drugs, postoperative analgesia, prevention and treatment of other outcomes, and obtaining maximum efficacy from the administered treatments. Further research is also needed to determine on how to develop further management strategies of these undesirable events.

## Figures and Tables

**Table 1:** Demographic and clinical characteristics of the participants.

Variables		Frequency	Percentage
Gender
	Male	259	55.1
	Female	211	44.9

Residence
	Urban	274	58.3
	Rural	196	41.7

Occupation
	Employed	235	50
	Unemployed	235	50

Hospital setting
	Halibet	173	36.8
	Orotta	297	63.2

Health coverage
	Paying	358	76.2
	Free	112	23.8

Type of anesthesia
	General	267	56.8
	Regional	203	43.2

Type of surgery
	General	261	55.5
	Orthopedic	99	21.1
	Gyn/obs	89	18.9
	ENT	7	1.5
	Burn	14	3.0

Admission type
	Emergency	109	23.2
	Elective	361	76.8

Age	**Mean**	**SD**	
	45.87	18.53	

**Table 2:** Prevalence of undesirable postoperative anesthesia outcomes (*n* = 470).

Discomfort and needs	Prevalence of UAO	
	At least a little bit	More than moderate
	*n* (%)	*n* (%)
Postoperative pain	388 (82.6)	205 (43.6)
Sore throat	87 (18.5)	10 (2.1)
Back pain	125 (26.6)	20 (4.3)
Nausea	191 (40.6)	44 (9.4)
Vomiting	177 (37.7)	47 (10)
Cold	194 (41.3)	80 (17)
Hunger	86 (18.3)	21 (4.5)
Thirst	130 (27.7)	47 (10)
Headache	118 (25.1)	25 (5.3)

Severity was graded as 0 = not at all; 1 = a little bit; 2 = moderately; 3 = quite a bit; 4 = extremely.

**Table 3:** Occurrence of undesirable postoperative anesthesia outcomes (types of surgery versus types of anesthesia).

	General surgery			Orthopedic surgery			Gyn/Obs surgery		
	GA	RA	OR (95% CI)	GA	RA	OR (95% CI)	GA	RA	OR (95% CI)
Postoperative pain	84.66	77.55	1.60 (0.85, 3.02)	75.68	77.42	0.91 (0.35, 2.37)	92	89.74	1.31 (0.31, 5.63)
Sore throat	26.38	6.12	5.49 (2.24, 13.46)^*∗∗∗*^	27.03	6.45	5.37 (1.54, 18.67)^*∗∗*^	24	10.27	2.76 (0.82, 9.37)
Back pain	17.79	30.61	0.49 (0.27, 0.88)^*∗*^	24.32	24.19	1.01 (0.39, 2.60)	30	56.41	0.33 (0.14, 0.80)^*∗*^
Nausea	49.08	35.71	1.74 (1.04, 2.90)^*∗*^	10.81	19.35	0.51 (0.15, 1.70)	60	64.1	0.84 (0.35, 2.00)
Vomiting	44.79	28.57	2.03 (1.19, 3.47)^*∗*^	16.22	16.13	1.01 (0.33, 3.04)	66	64.1	1.09 (0.45, 2.62)
Cold	44.17	40.82	1.15 (0.70, 1.91)	27.03	32.26	0.79 (0.32, 1.91)	44	58.97	0.55 (0.23, 1.28)
Hunger	20.25	20.41	0.99 (0.53, 1.85)	2.7	14.52	0.16 (0.02, 1.35)	24	17.95	1.44 (0.51, 4.10)
Thirst	33.13	30.61	1.12 (0.66, 1.93)	8.11	12.9	0.60 (0.15, 2.40)	36	33.33	1.13 (0.47, 2.72)
Headache	18.4	28.57	0.56 (0.31, 1.02)	27	27.42	0.98 (0.39, 2.45)	20	38.46	0.40 (0.16, 1.03)

RA (regional anesthesia)-reference category. ^*∗*^*p* < 0.05, ^*∗∗*^*p* < 0.01, ^*∗∗∗*^*p* < 0.001.

## Data Availability

The datasets generated and/or analyzed during the current study are available from the corresponding author upon request.

## Abbreviations

CI: Confidence interval
ENT: Ear, nose, and throat
GA: General anesthesia
Gyn/Obs: Gynecology and Obstetrics
LPPSq: Leiden Perioperative care Patient Satisfaction questionnaire
NSAIDs: Nonsteroidal anti-inflammatory drugs
OR: Odds ratio
POD: Postoperative day
PONV: Postoperative nausea and vomiting
RA: Regional anesthesia
SD: Standard deviation
SPSS: Statistical Package for the Social Sciences
UAO: Undesirable anesthesia outcomes.
